# Supplementary material for: Phytochemical Characterisation and Antioxidant and Anti-Inflammatory Potential of Muscari neglectum (Asparagaceae) Bulbs
Source: Molecules. 2025 Nov 10;30(22):4351. doi: 10.3390/molecules30224351 (PMC12654854; doi:10.3390/molecules30224351)
Supplement: Supplementary file 1 [file molecules-30-04351-s001.zip › molecules-3923436-supplementary.pdf]

Supplementary Material

Phytochemical Characterisation and Antioxidant and Anti-Inflammatory Potential of *Muscari neglectum* (Asparagaceae) Bulbs

María del Carmen Villegas-Aguilar [1], Antonio Segura-Carretero [1,*] and Víctor N. Suárez-Santiago [2,*]

[1] Department of Analytical Chemistry, University of Granada, 18071 Granada, Spain; marivillegas@ugr.es
[2] Department of Botany, University of Granada, 18071 Granada, Spain
* Correspondence: ansegura@ugr.es (A.S.-C.); vsuarez@ugr.es (V.N.S.-S.); Tel.: +34-958248435 (A.S.-C.); +34-958248814 (V.N.S.-S.)

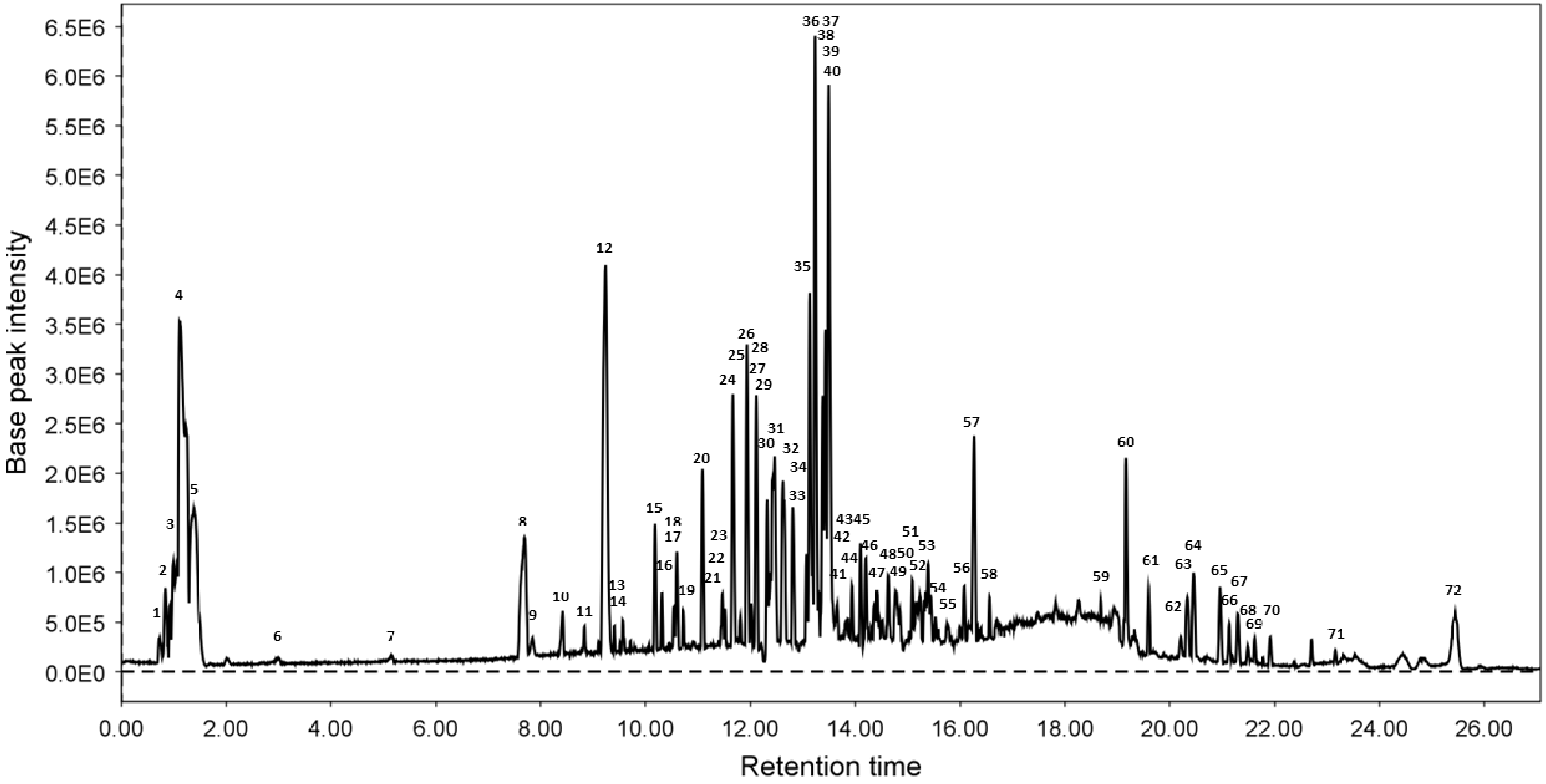

Figure S1. Base peak chromatogram from the *Muscari neglectum* bulb extract.

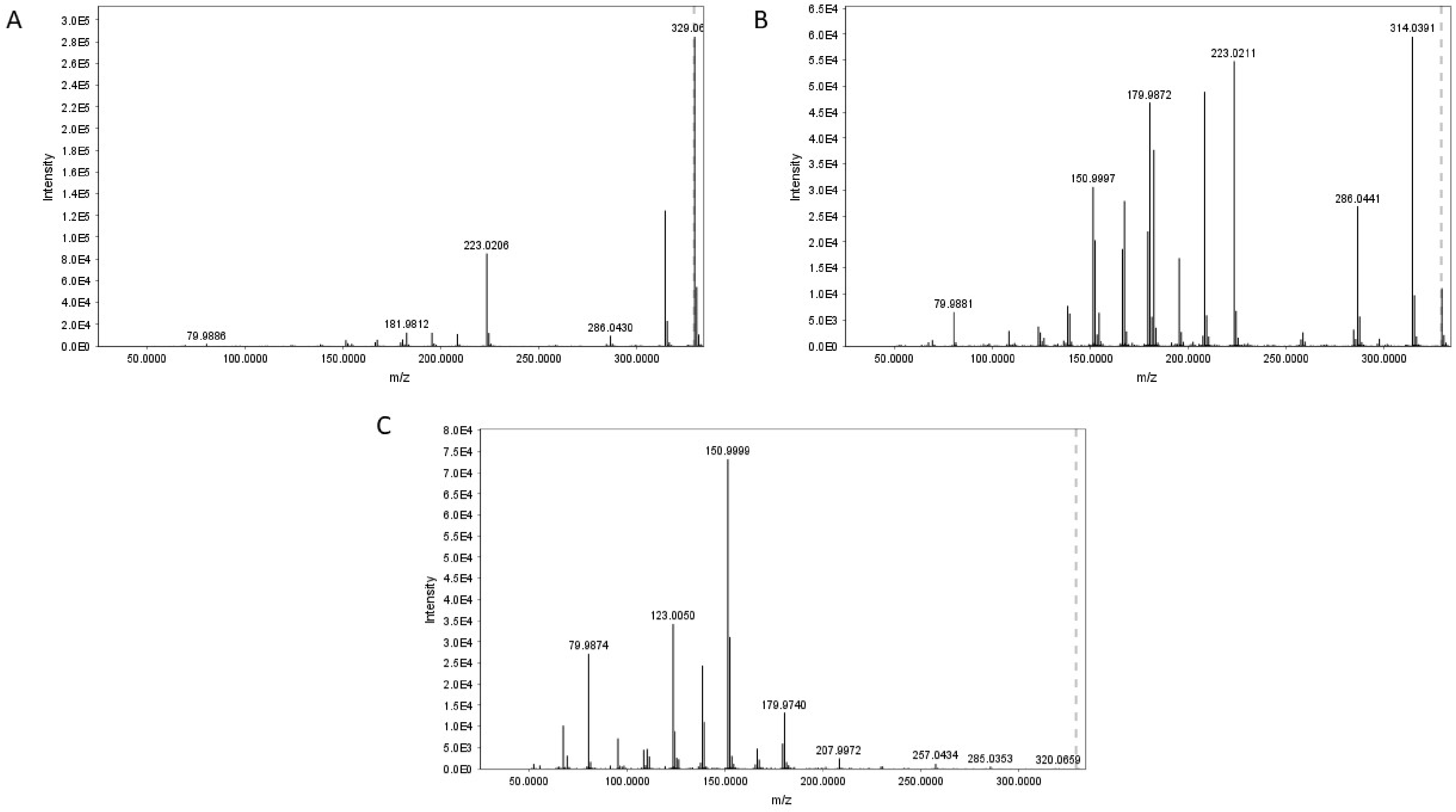

Figure S2. Mass fragmentation spectrum of Dimethylquercetin (m/z: 329.0645). A: collision energies: 10 eV. B: collision energies: 20 eV. C: collision energies: 40 eV.

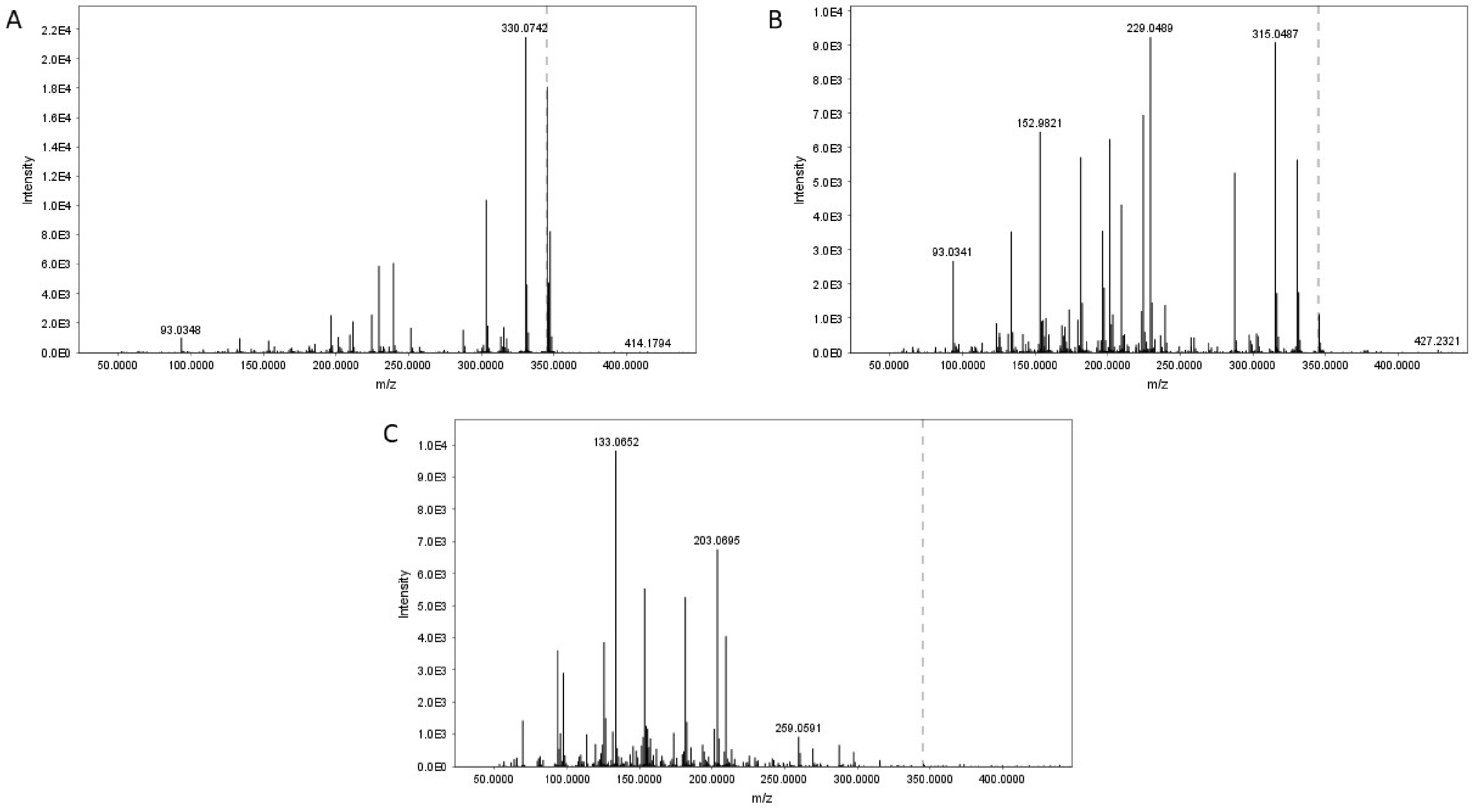

Figure S3. Mass fragmentation spectrum of Muscomin isomer 1 (m/z: 345.0983). A: collision energies: 10 eV. B: collision energies: 20 eV. C: collision energies: 40 eV.

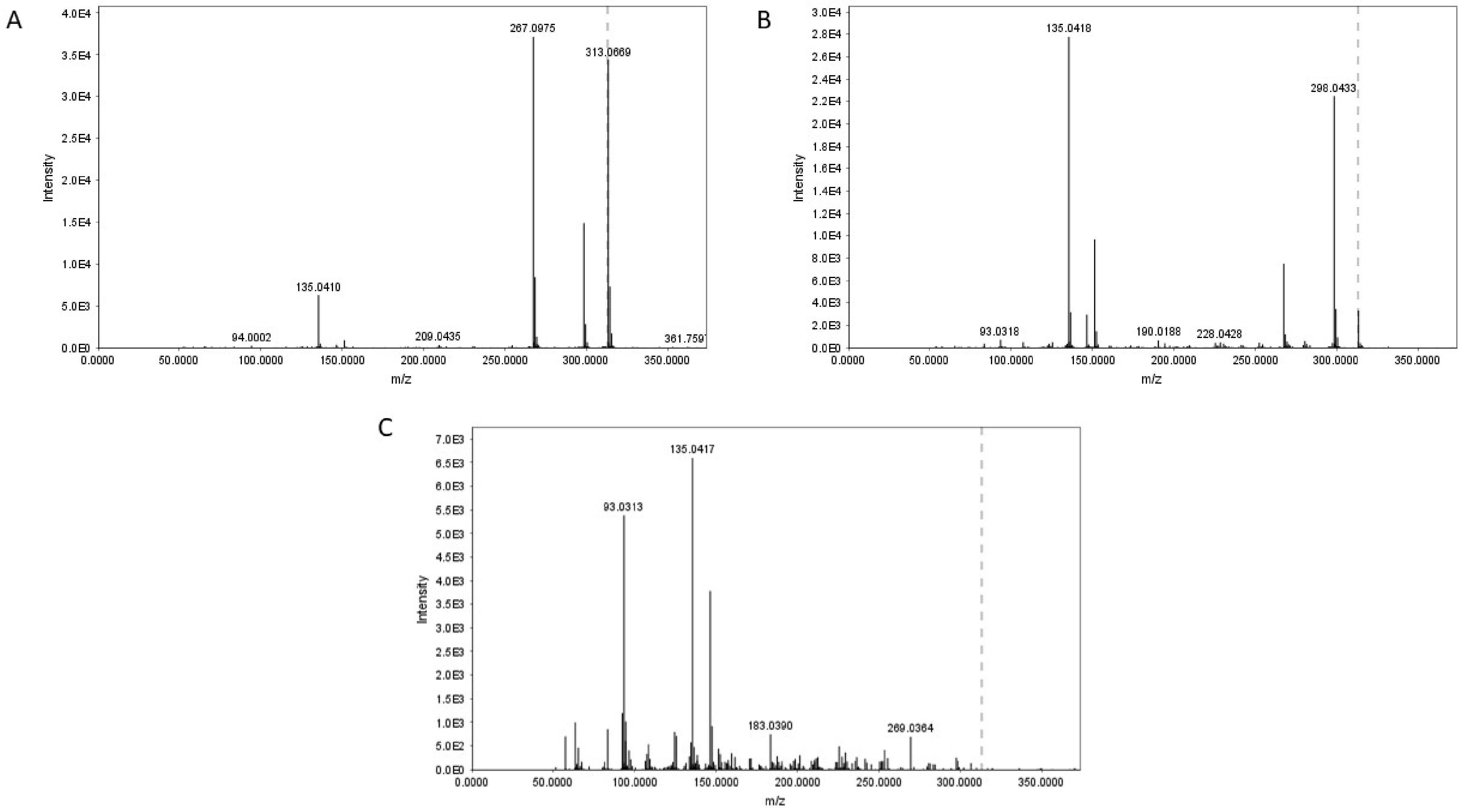

Figure S4. Mass fragmentation spectrum of Muscomosin isomer 1 (m/z: 313.0722). A: collision energies: 10 eV. B: collision energies: 20 eV. C: collision energies: 40 eV.

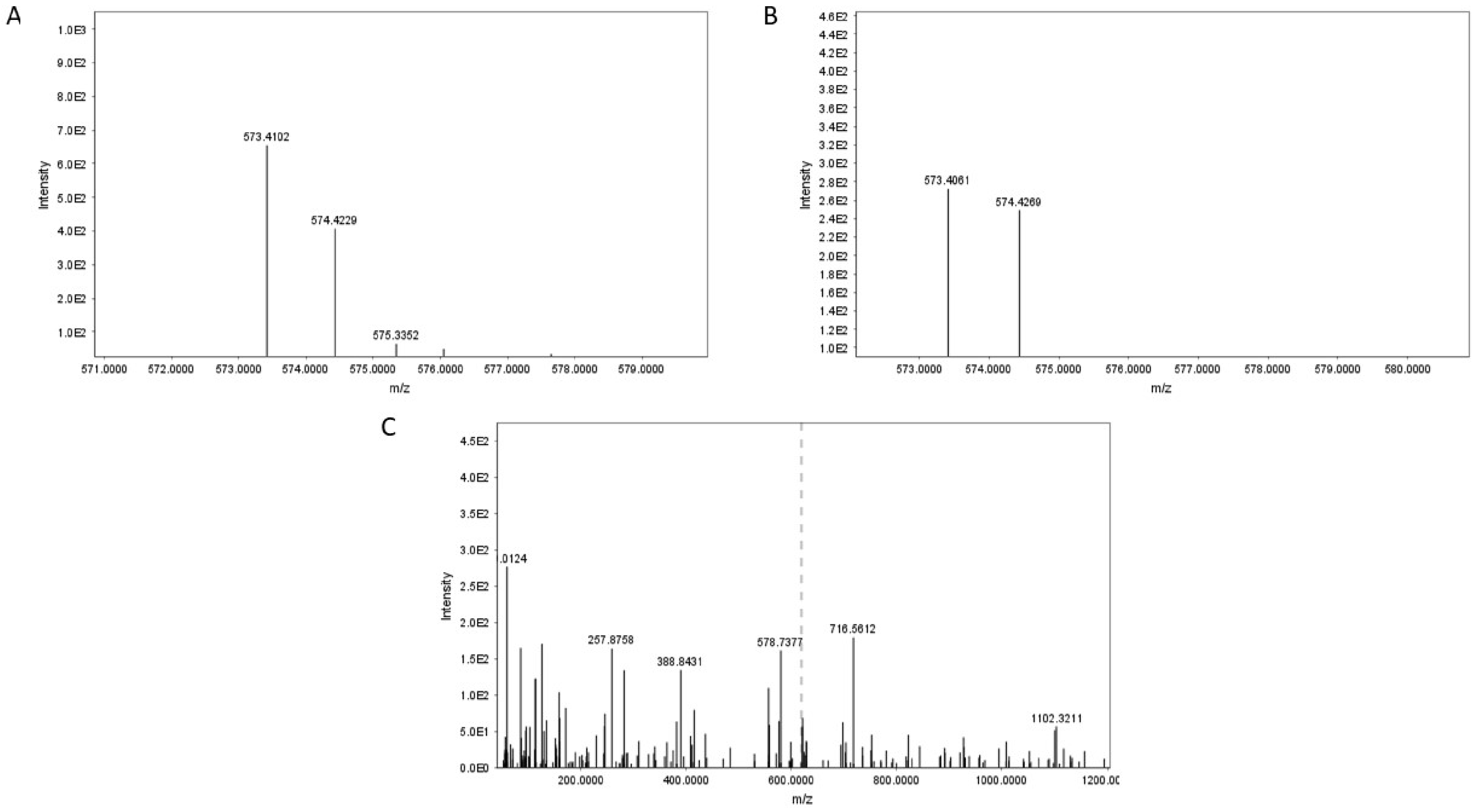

Figure S5. Mass fragmentation spectrum of Ginsenoside Rh4 isomer 1 (m/z: 619.4217). A: collision energies: 10 eV. B: collision energies: 20 eV. C: collision energies: 40 eV.

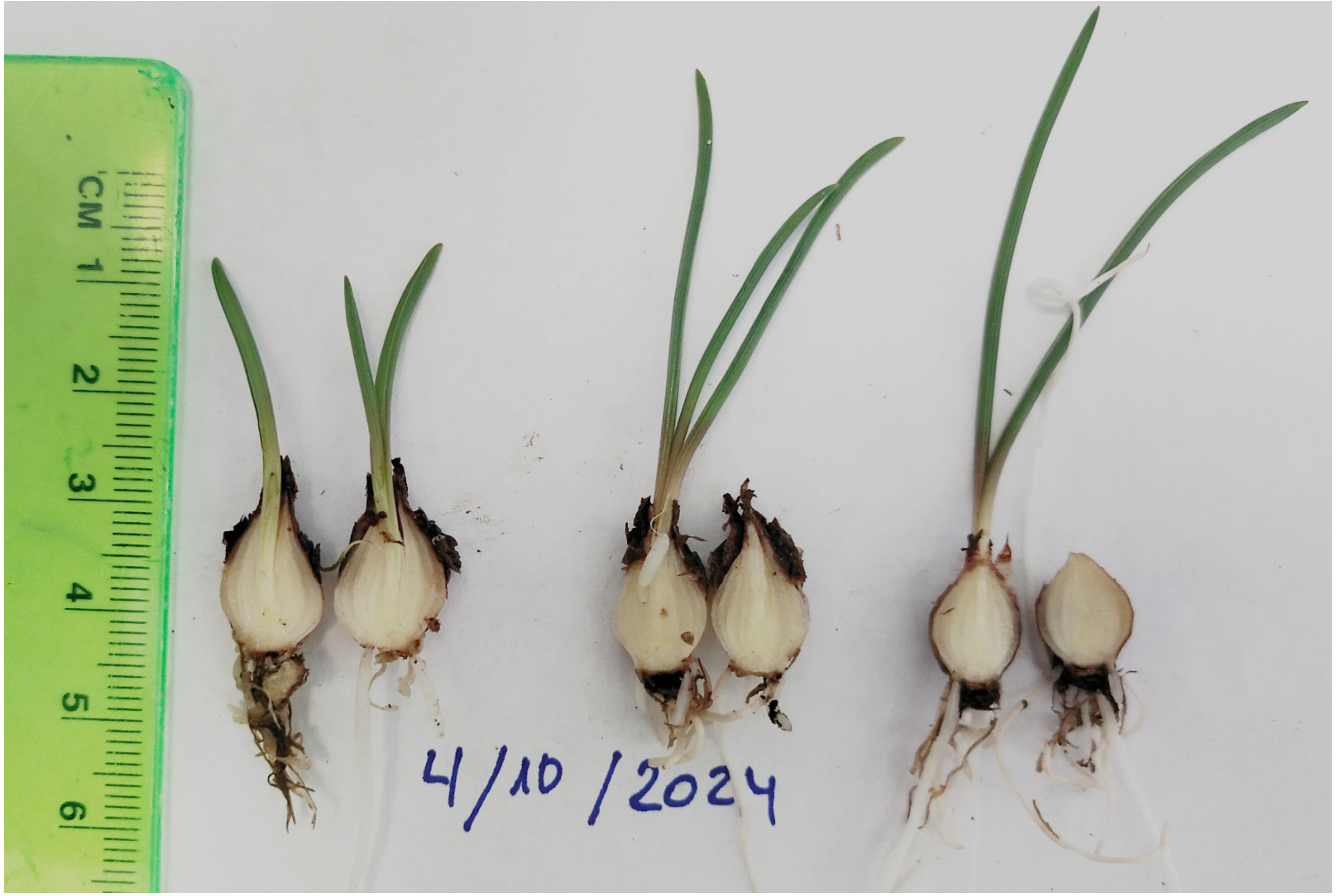

Figure S6. Developmental stage of *Muscari neglectum* bulbs at the time of collection.
